# Draft genome sequence of uropathogenic *Escherichia coli* U13824, a multidrug-resistant (MDR) and extended-spectrum-β-lactamase (ESBL)-producing UPEC strain isolated from an adult woman with urinary tract infection

**DOI:** 10.1128/mra.00027-24

**Published:** 2024-05-21

**Authors:** José Antonio Magaña-Lizárraga, Bruno Gómez-Gil, Julisa Enciso-Ibarra, Yesenia Sánchez-Lugo, Jesús Ricardo Parra-Unda, José Trinidad Rodríguez-Atondo, Saúl Beltrán-Fernández, María Elena Báez-Flores

**Affiliations:** 1 Unidad de Investigaciones en Salud Pública “Dra. Kaethe Willms,” Facultad de Ciencias Químico-Biológicas, Universidad Autónoma de Sinaloa, Culiacán, Sinaloa, Mexico; 2 Centro de Investigación en Alimentación y Desarrollo, A.C. (CIAD), Unidad Mazatlán en Acuicultura y Manejo Ambiental, Mazatlán, Sinaloa, México; 3 Unidad de Farmacovigilancia y Tecnovigilancia, Hospital General de Culiacán “Bernardo J. Gastélum,” Culiacán, Sinaloa, México; 4 Laboratorio de Análisis Clínicos, Hospital General de Culiacán “Bernardo J. Gastélum,” Culiacán, Sinaloa, México; 5 Centro de Investigación Epidemiológica de Sinaloa, Hospital General de Culiacán “Bernardo J. Gastélum,” Culiacán, Sinaloa, México; University of Maryland School of Medicine, Baltimore, Maryland, USA

**Keywords:** *E. coli*, MDR ESBL producing, uropathogenic *E. coli*, antimicrobial resistance

## Abstract

Urinary tract infections (UTIs) caused by multidrug-resistant and extended-spectrum β-lactamase-producing uropathogenic *Escherichia coli* are a worldwide concern. We report the draft genome of *E. coli* U13824 isolated from a female outpatient with UTI. This genome’s availability strengthens the genomic surveillance of antimicrobial resistance and the spreading of these strains.

## ANNOUNCEMENT

Urinary tract infections (UTIs) are a significant cause of human infections with over 150 million cases annually. The primary etiological agent is uropathogenic *Escherichia coli* (UPEC), of which multidrug-resistant (MDR) extended-spectrum β-lactamase (ESBL) producer strains are globally increasing (1, 2). ESBL-producing *E. coli* is among the top-priority pathogens listed by the World Health Organization (3, 4). Global genomic surveillance for these MDR pathogens is essential for public health intelligence, allowing disease control strategies given the limited options for effective treatment (5). We report the MDR ESBL-producing *E. coli* U13824 genome, isolated from a female UTI case.

*E. coli* U13824 was derived from a midstream-voided urine specimen collected in 2019 from a 57-year-old female outpatient at the General Hospital of Culiacan, Sinaloa, Mexico. Ethical approval was granted by the Research Ethics Committee of the General Hospital (CONBIOÉTICA-25-CEI-001-20160708). The urine sample was cultured on MacConkey agar and incubated for 24 h at 37°C. The hospital’s clinical laboratory determined the bacterial identity and the antibiotic-resistance profile using the MicroScan WalkAway 96 Plus System (Beckman Coulter Inc., USA).

Genomic DNA (gDNA) was extracted from a single colony cultured overnight in Luria-Bertani broth at 37°C employing the Wizard Genomic DNA Purification Kit (Promega Corporation, Madison, WI, USA) and quantified by Quibit 2.0 Fluorometer (Thermo Fisher Scientific, Massachusetts, USA). The genomic library was prepared using the Nextera XT DNA Library Preparation Kit (Illumina, San Diego, CA, USA), followed by paired-end sequencing (2 × 150 bp) on the Illumina MiniSeq Instrument (300 cycles) yielding 1,879,726 pairs of reads. Raw reads were examined using FastQC v0.11.9 (6) and processed with Cutadapt v2.4 (7) to remove adapter sequences, low-quality bases (<*Q*30), and short reads (<50 bp). Genome assembly was performed with SPAdes v3.15.1 (8) ( --careful and -k 21,33,55,77,99,107,117 options), followed by a reference-guided scaffolding with MeDuSa v1.6 (9), using *E. coli* Ecol_AZ146 (GCF_002012085.1) as reference, and excluding scaffolds shorter than 300 bp. The annotation was accomplished through the NCBI Prokaryotic Genome Annotation Pipeline v6.6 (10). The phylogenetic group was determined using ClermonTyping v21.03 (http://clermontyping.iame-research.center/) (11). The multilocus sequence typing (MLST), serotyping, acquired antimicrobial resistance genes (ARGs), and virulence-associated genes (VAGs) searching were performed by MLST v2.0.9 (12), SerotypeFinder v2.0.1 (13), ResFinder v4.1 (14, 15), and VirulenceFinder v2.0.3 (16, 17), respectively (Center for Genomic Epidemiology, https://www.genomicepidemiology.org/), setting threshold parameters of 90% identity and 60% coverage. Default parameters were used except where otherwise noted.

The assembly resulted in 26 scaffolds with a scaffold N_50_ value of 5.3 Mb and an average coverage depth of 43×. The genome size was 5,369,689 bp in length, 50.71% of GC content, and genome completeness of 98.89%. The gene prediction identified 5,442 genes, of which 5,077 were protein-coding sequences, 278 pseudogenes, 75 tRNAs, 7 RNAs, and five ncRNAs. The *in silico* analyses determined *E. coli* U13824 belongs to the B2-O25:H4-ST2279 subclone. Table 1 describes the acquired ARGs and VAGs identified in *E. coli* U13824, highlighting the co-harboring of β-lactamase-encoding genes and VAGs related to ExPEC and UPEC pathovars (18–21).

**TABLE 1:** Antimicrobial resistance and virulence determinants of the *E. coli* strain U13824 isolated from female UTI

Strain ID	Acquired antimicrobial resistance genes	QRDR mutations^*a*^	Virulence genes^*b*^
U13824	*aac(6')-Ib-cr*; *aac (3)-IIa*; *aph(3'')-Ib*; *aph (6)-Id*; *bla_CTX-M-15_*; *bla_TEM-1B_*; *bla_OXA-1_*; *catB3*; *tet(A*); *tet(B*); *dfrA8*; *sul2*; *sitABCD*	*gyrA p.S83L gyrA p.D87N parC p.S80I parC p.E84V parE p.I529L*	*capU*; *chuA*; *cnf1*; *fyuA*; *gad*; *hra*; *iha*; *irp2*; *iss*; *iucC*; ***iutA***; *kpsE*; ***kpsMII_K5***; *ompT*; ***papA_F43***; ***papC***; *sat*; *sitA*; *terC*; *traT*; *usp*; *yfcV*

^*a*^ QRDR, quinolone resistance-determining region.

^*b*^ ExPEC-associated virulence genes by Johnson et al. (19) are highlighted in bold. UPEC-associated virulence genes by Spurbeck et al. (20) and Nipič et al. (21) are underlined.

## Data Availability

The Whole-Genome Shotgun (WGS) project of *Escherichia coli* U13824 has been deposited in DDBJ/ENA/GenBank under BioProject accession number PRJNA715781. The raw sequence data were deposited in the Sequence Read Archive (SRA) under the accession number SRR21010776, Assembly accession number ASM3384336v1, BioSample accession number SAMN30247026, and genome sequence accession number JAWXVL000000000. The version described in this paper is the first version, JAWXVL010000000.
